# Different Sex-Based Responses of Gut Microbiota During the Development of Hepatocellular Carcinoma in Liver-Specific *Tsc1*-Knockout Mice

**DOI:** 10.3389/fmicb.2018.01008

**Published:** 2018-05-16

**Authors:** Rong Huang, Ting Li, Jiajia Ni, Xiaochun Bai, Yi Gao, Yang Li, Peng Zhang, Yan Gong

**Affiliations:** ^1^Department of Hepatobiliary Surgery II, Guangdong Provincial Research Center of Artificial Organ and Tissue Engineering, Zhujiang Hospital of Southern Medical University, Guangzhou, China; ^2^State Key Laboratory of Organ Failure Research, Southern Medical University, Guangzhou, China; ^3^Department of Cell Biology, School of Basic Medical Science, Southern Medical University, Guangzhou, China

**Keywords:** biomarker, gut microbiota, hepatocellular carcinoma, sex-based response, *Tsc1*-knockout mice

## Abstract

Gut microbial dysbiosis is correlated with the development of hepatocellular carcinoma (HCC). Therefore, analyzing the changing patterns in gut microbiota during HCC development, especially before HCC occurrence, is essential for the diagnosis and prevention of HCC based on gut microbial composition. However, these changing patterns in HCC are poorly understood, especially considering the sex differences in HCC incidence and mortality. Here, with an aim to determine the relationship between gut microbiota and HCC development in both sexes, and to screen potential microbial biomarkers for HCC diagnosis, we studied the changing patterns in the gut microbiota from mice of both sexes with liver-specific knockout of *Tsc1* (*LTsc1KO*) that spontaneously developed HCC by 9–10 months of age and compared them to the patterns observed in their wide-type *Tsc1^fl/fl^* cohorts using high-throughput sequencing. Using the *LTsc1KO* model, we were able to successfully exclude the continuing influence of diet on the gut microbiota. Based on gut microbial composition, the female *LTsc1KO* mice exhibited gut microbial disorder earlier than male *LTsc1KO* mice during the development of HCC. Our findings also indicated that the decrease in the relative abundance of anaerobic bacteria and the increase in the relative abundance of facultative anaerobic bacteria can be used as risk indexes of female HCC, but would be invalid for male HCC. Most of the changes in the gut bacteria were different between female and male *LTsc1KO* mice. In particular, the increased abundances of *Allobaculum*, Erysipelotrichaceae, Neisseriaceae, *Sutterella*, Burkholderiales, and *Prevotella* species have potential for use as risk indicators of female HCC, and the increased abundances of Paraprevotella, Paraprevotellaceae, and *Prevotella* can probably be applied as risk indicators of male HCC. These relationships between the gut microbiota and HCC discovered in the present study may serve as a platform for the identification of potential targets for the diagnosis and prevention of HCC in the future.

## Introduction

Recent research on gut microbiota has changed our understanding about their role in human diseases and their potential medical impact (Qin et al., 2012). From the perspective of both taxonomic and functional composition, gut microbiota might be linked to and contribute to many complex diseases such as obesity (Turnbaugh et al., 2009; Zhang et al., 2009; Fei and Zhao, 2013), type 2 diabetes (Larsen et al., 2010; Qin et al., 2012), and Crohn’s disease (Manichanh et al., 2006; Joossens et al., 2011).

Due to the anatomic connection, the liver is constantly exposed to microbial products from the gut microbiota (Zhang et al., 2012). Therefore, disruption of gut homeostasis (dysbiosis) is associated with numerous liver diseases (Schnabl, 2013; Xie et al., 2016) such as alcoholic fatty liver disease (Adachi et al., 1995), nonalcoholic fatty liver disease (Li et al., 2003), liver fibrosis (Seki and Schnabl, 2012), and liver cirrhosis (Karakan, 2014; Qin et al., 2014).

Such dysbiosis is also associated with the development of hepatocellular carcinoma (HCC, Zhang et al., 2012), which is the most frequent and aggressive primary tumor of the liver and has limited treatment options (El-Serag and Rudolph, 2007; Sherman, 2010; Shao et al., 2017). Lipopolysaccharide (LPS, i.e., endotoxins) is a major component of the outer membrane of gram-negative bacteria; consequently, an increase in the relative abundance of gram-negative bacteria in the gut microbiota could increase the concentrations of endotoxins in the plasma and the liver (Xie et al., 2016). Several lines of evidence indicate that LPS accumulation contributes to the pathogenesis of HCC by eliciting proinflammatory responses in the liver (Yu et al., 2010).

These data have led to a growing interest in gut microbiota and their metabolites as a new therapeutic target for the prevention, diagnosis, and treatment of metabolic diseases including HCC (Jia et al., 2008; Cani and Delzenne, 2011; Rajpal and Brown, 2013). However, despite considerable progress, most studies have been focused on comparing differences in gut microbiota between patients with HCC and healthy controls. Although differences in phylogenetic and functional compositions have been found, it cannot be determined whether these differences are an inducing factor or a consequence of HCC, only through comparison of the differences between patients with HCC and healthy controls. A better understanding of how variations in the symbiotic supraorganism contribute to HCC risk sustainability during HCC development and progression will point the way to new therapeutic interventions and HCC prevention strategies (Nicholson et al., 2012). Therefore, the phylogenetic and functional compositions of gut microbiota associated with HCC development and progression deserve more attention. More research focused on obtaining detailed information about changes in gut microbial composition and their roles in HCC development and progression is also needed.

Previously, Xie et al. (2016) analyzed fecal microbiota at various pathological stages in the liver and found gut microbiota to be significantly altered in the progression of liver disease. However, the authors did not exclude the continuing influence of streptozotocin-high fat diet (STZ-HFD) on the gut microbiota, despite diet being a major factor that casts and changes the gut microbiota (Faith et al., 2011; Claesson et al., 2012). However, Menon et al. (2012) reported that mice with liver-specific knockout of *Tsc1*(*LTsc1KO*) developed spontaneous HCC by 9–10 months of age. In this *LTsc1KO* model, tumor development was preceded by all of the hallmarks of HCC and was initiated by hepatocyte damage (Menon et al., 2012). This study provided an appropriate model to investigate the causality between HCC and gut microbiota disorder, which excludes the influence of diet and other inducers. We predicted that the gut microbiota from the *LTsc1KO* mice gradually divided to those from the wild types accompanying their growth and gram-negative bacteria were elevated in the gut microbiota of the *LTsc1KO* mice. The increase in the abundance of gram-negative bacteria resulted in increased endotoxin levels and triggered liver inflammation and other factors that induce HCC. In the present study, to evaluate the changes occurring in the gut microbiota of *LTsc1KO* mice with growth, the gut microbiota of *LTsc1KO* mice and their wide-type *Tsc1^fl/fl^* cohorts at different ages were investigated using MiSeq high-throughput sequencing of the 16S rRNA gene. Our data provide insights into the characteristics of gut microbiota related to the development of HCC, provide a paradigm for future studies on the pathophysiological roles of gut microbiota in other relevant diseases, and highlight the potential usefulness of a gut microbiota-based approach for the assessment of individuals at risk for HCC.

## Materials and Methods

### Animal Studies

Adult *LTsc1KO* mice and their wide-type *Tsc1^fl/fl^* cohorts were purchased from the Jackson Laboratory (Bar Harbor, ME, United States). All the mice were housed in individual stainless-steel cages, provided water *ad libitum*, and fed a nutritionally replete SPF solid diet that contained ≤100 g of moisture, ≥200 g of crude protein, ≥40 g of crude fat, ≤50 g of crude fiber, and ≤80 g of crude ash per kg. The male and female mice used in this study were propagated using adult *LTsc1KO* and *Tsc1^fl/fl^* mice. The mice were subdivided into five groups according to their age: group 1, age: 68–75 days (seven and six individuals of *LTsc1KO* and *Tsc1^fl/fl^* mice, respectively); group 2, age: 100–133 days (six and six individuals of *LTsc1KO* and *Tsc1^fl/fl^* mice, respectively); group 3, age: 171–172 days (six and five individuals of *LTsc1KO* and *Tsc1^fl/fl^* mice, respectively); group 4, age: 185–191 days (seven and five individuals of *LTsc1KO* and *Tsc1^fl/fl^* mice, respectively); and group 5, age: 222–322 days (six and four individuals of *LTsc1KO* and *Tsc1^fl/fl^* mice, respectively) (Supplementary Table S1). The animal experiments were approved by the Animal Ethics Committee of Southern Medical University under the approval number SYXK(Yue)2011-0074 and performed in accordance with animal ethics guidelines and approved protocols.

### Measurement of Physical Indexes and Observation of Liver Histology

The mice were anesthetized using 1% of carbrital and their blood was collected from the heart. The blood was placed for 2 h at room temperature before centrifugation for 3 min at 1370 *g* for collecting the serum. The serum concentrations of alanine aminotransferase (ALT) and aspartate aminotransferase (AST) were measured using an animal dry biochemical automatic analyzer (Catalyst One, United States). Liver histological analysis was conducted by dissecting the anesthetized mice and observing sections of paraffin-embedded, formalin-fixed tissues stained with hematoxylin and eosin (Yang et al., 1997).

### Fecal Sample Collection and High-Throughput Sequencing

Approximately 0.1 g of fresh fecal pellets was collected from each mouse and stored at -20°C for microbial DNA extraction. Fecal microbial DNA was extracted using a PowerSoil^TM^ DNA isolation kit (MO BIO, United States). DNA concentration and quality were checked using a NanoDrop spectrophotometer (Thermo Fisher Scientific, United States).

The V4-V5 hypervariable region of 16S rRNA gene was amplified and sequenced using the MiSeq system, as described previously (Li et al., 2016; Ni et al., 2017). The sequences were processed and alpha-diversities were calculated using the QIIME Pipeline 1.9.0 with default parameters (Caporaso et al., 2010). Chimeric sequences were identified and removed using the Uchime algorithm before further analysis (Edgar et al., 2011). To assess the repeatability of the MiSeq sequencing in microbiota analysis, the samples were sequenced at least two times. Samples with less than 5000 sequences were removed and then 5000 sequences were randomly sampled from each residual sample to calculate the weighted UniFrac dissimilarity, perform procrustes analysis (Muegge et al., 2011; Rampelli et al., 2013), and employ the unweighted pair group method with arithmetic means (UPGMA). The sequences from the same sample were merged as the sequence dataset of the corresponding sample for further analysis. Subsequently, all the samples were randomly re-sampled to obtain the same number of sequences. The high-quality sequences were clustered into operational taxonomic units (OTUs) at a 97% identity using UPARSE (Edgar, 2013). Taxonomic assignments of each OTU were determined using the RDP classifier (Wang et al., 2007). The metabolic characteristics, ratio of gram-negative bacteria to gram-positive bacteria, and functional profiles of the fecal microbiota were calculated using BugBase (Ward et al., unpublished) and PICRUSt (Langille et al., 2013).

All the DNA datasets have been submitted to the NCBI Sequence Read Archive database under the accession number SRP107826.

### Statistical Analysis

Results for each parameter are presented as mean ± standard error (SE) for each group. Non-parametric multivariate analysis of variance (MANOVA) (Anderson, 2001) that was used to test the different significance among three and more groups was conducted using the R vegan package (Dixon, 2003). Canonical correspondence analysis (CCA) was conducted using the R vegan package and ade4 package. The Wilcoxon rank sum test was used to test significant difference between two groups. Tukey’s method was used to perform multiple comparisons of means. *P*-values ≤ 0.05 were considered statistically significant.

## Results

### HCC Development of *LTsc1KO* Mice

**FIGURE 1:**
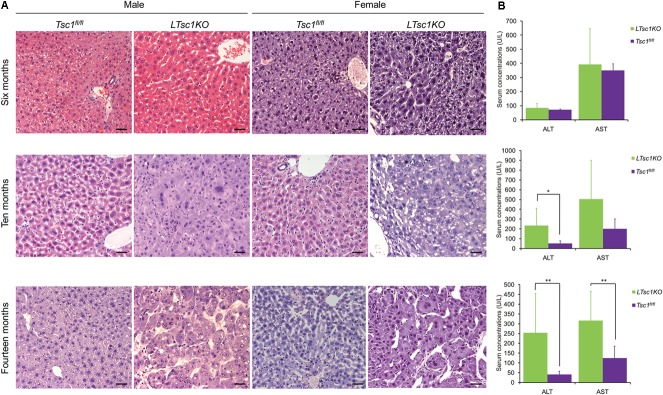
Hepatocellular carcinoma (HCC) development in the *LTsc1KO* mice. **(A)** Representative hematoxylin and eosin-stained sections of the livers of the *Tsc1^fl/fl^* and *LTsc1KO* mice. **(B)** Serum concentrations of alanine aminotransferase (ALT) and aspartate aminotransferase (AST) are presented as the means ± standard error of the mean (SEM). ^∗^*P* < 0.05 and ^∗∗^*P* < 0.01. Scale bars, 50 μm (×200).

Consistent with a previous report (Menon et al., 2012), we found no detectable tumors in any of the *LTsc1KO* mice in postnatal 6 months. However, Menon et al. (2012) reported that, at this stage (postnatal 6 months), the livers of all the mice had shown various characteristics of liver damage, including increased serum concentrations of the liver enzymes ALT and AST. In contrast, no significant difference was detected in the serum concentrations of AST and ALT between the *LTsc1KO* and *Tsc1^fl/fl^* mice at postnatal 6 months in the present study (Wilcoxon rank sum test, *P* > 0.05). However, at 10–14 months of age, an increase in the serum concentrations of the liver enzymes ALT and AST was observed in the *LTsc1KO* mice (**Figure 1**). Liver histology with hematoxylin and eosin staining showed no significant pathological change in the livers of the 6-month-old *LTsc1KO* mice. However, HCC was detected at 10 and 14 months of age. No pathological change was detected in the livers of the *Tsc1^fl/fl^* mice at any age (**Figure 1**).

### Repeatability of the Results Obtained Using Sequencing Technology to Analyze Fecal Microbiota

**FIGURE 2:**
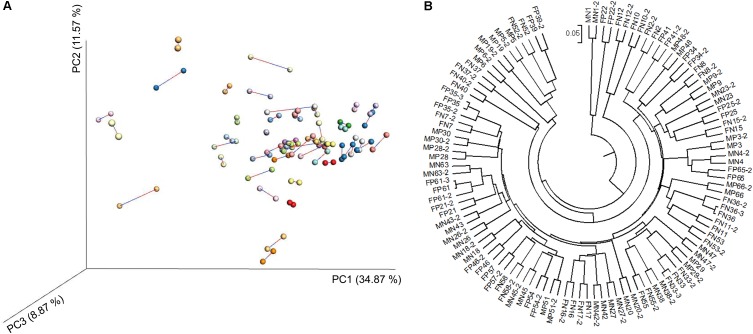
Principal coordinate analysis (PCoA) profile of procrustes analysis **(A)** and unweighted pair group method with arithmetic means (UPGMA) profile **(B)** based on the weighted UniFrac dissimilarity matrix of fecal microbiota samples from *LTsc1KO* and *Tsc1^fl/fl^* mice. The *LTsc1KO* mice are a genetic mouse model with liver-specific knockout of the *Tsc1* gene, which causes them to develop spontaneous HCC by 9–10 months of age, and the *Tsc1^fl/fl^* mice are their wide-type cohorts.

We carried out high-throughput sequencing of the 16S rRNA gene in individual fecal DNA samples from 58 mice (26 *LTsc1KO* mice and 32 *Tsc1^fl/fl^* controls, Supplementary Table S1). After removing samples with less than 5000 sequences from single sampling, 54 fecal samples remained and were used to verify the repeatability of the results obtained using the sequencing technology. Despite using only 5,000 sequences of each specimen, MiSeq sequencing still exhibited a forceful repeatability for each sample (**Figure 2**).

### Changes in the Gut Microbiota Between *LTsc1KO* and Wide-Type *Tsc1^fl/fl^* Mice at Different Ages

After merging the sequences from the same sample, a total of 1,703,063 high-quality sequences were obtained. To eliminate the influence of sequencing depth, 15,885 sequences were randomly sampled from each sample for further analysis. A total of 291,336 OTUs from 498 genera were identified by grouping sequences at the 97% similarity level. Although the alpha diversity of the gut microbiota from the *LTsc1KO* and *Tsc1^fl/fl^* mice fluctuated during the experiment (Supplementary Figure S1), no significant difference was detected between the *LTsc1KO* and *Tsc1^fl/fl^* mice.

**FIGURE 3:**
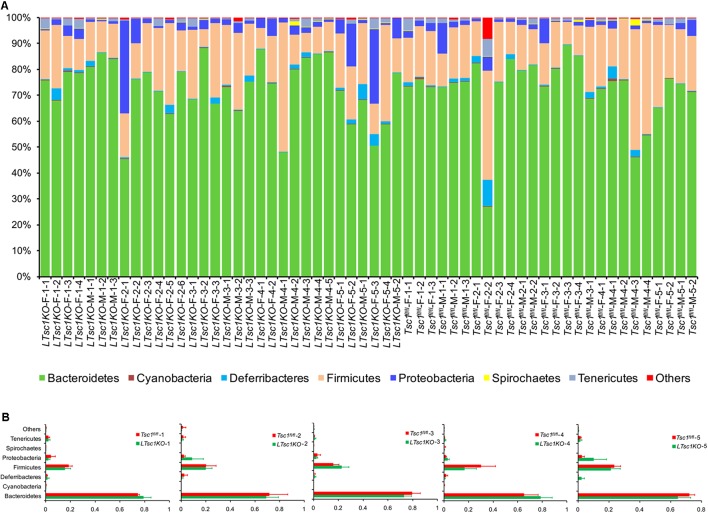
Percentage bar diagram **(A)** and composite bar diagram **(B)** showing compositions of the dominant phyla in fecal microbiota from *LTsc1KO* and *Tsc1^fl/fl^* mice. *LTsc1KO* mice are a genetic mouse model with liver-specific knockout of the *Tsc1* gene, which causes them to develop spontaneous HCC by 9–10 months of age, and the *Tsc1^fl/fl^* mice are their wide-type *Tsc1^fl/fl^* cohorts. The first numbers in the sample names indicate the group number. Group 1–5 contained mice of postnatal ages of 68–75, 100–133, 171–172, 185–191, and 222–322 days, respectively. The second numbers in the sample names indicate the individual numbers of the mice in each group.

The sequences were found to belong to 36 phyla with the exception of tiny unclassified sequences (0.019–6.29%). However, only seven phyla — Bacteroidetes, Firmicutes, Proteobacteria, Deferribacteres, Tenericutes, Spirochaetes, and Cyanobacteria — dominated the fecal microbial communities (their relative abundances were more than 1% in at least one sample; **Figure 3A**). No significant difference was detected between the gut microbiota from the *LTsc1KO* and *Tsc1^fl/fl^* mice of the same age (**Figure 3B**). However, this result contradicted the results of two previous studies that indicated that, with advancing cirrhosis, there is an increase in the abundance of Proteobacteria, the phylum containing gram-negative families such as Enterobacteriaceae (Chen et al., 2011; Bajaj et al., 2012).

Aerobic gram-negative bacteria in the gut microbiota have been found to be the major source of serum endotoxin (Riordan and Williams, 2006). Therefore, we compared the relative abundance of aerobic gram-negative bacteria in the fecal microbiota of the *LTsc1KO* and *Tsc1^fl/fl^* mice of different ages. Different changing patterns were detected between female and male mice at different ages (Supplementary Figure S2). The relative abundance of aerobic bacteria in the *LTsc1KO* mice in group 5 was obviously higher than that in the *Tsc1^fl/fl^* mice among the females, but no significant difference was found in the male mice (Supplementary Figure S2A). In contrast, the relative abundance of anaerobic bacteria was obviously decreased in the female *LTsc1KO* mice in group 3 compared with that in the corresponding female *Tsc1^fl/fl^* cohorts. Such obviously decreases were also observed in male *LTsc1KO* mice in groups 1 and 5 (compared with that in male *Tsc1^fl/fl^* mice; Supplementary Figure S2B). An increase in the abundance of facultative anaerobic bacteria in the gut microbiota is commonly considered to be associated with HCC (Zhang et al., 2012). However, we did not find any significant changes in the relative abundance of facultative anaerobic bacteria between the *LTsc1KO* and *Tsc1^fl/fl^* mice in any of the groups, except in group 3, where it was obviously increased in female *LTsc1KO* mice compared with that in corresponding female *Tsc1^fl/fl^* mice (Supplementary Figure S2C). Moreover, no significant difference was detected between the relative abundance of gram-negative or gram-positive bacteria from the *LTsc1KO* and *Tsc1^fl/fl^* mice in any of the groups (Supplementary Figures S2D,E). For LPS metabolism, no significant difference was detected in the relative abundance of the genes that participate in the KEGG pathways of LPS biosynthesis, LPS transport system, or LPS export system between the *LTsc1KO* and *Tsc1^fl/fl^* mice in any of the groups (Supplementary Figures S2F–H). Together, these results implied that gut microbiota of female mice were more sensitive to liver injury than those of male mice. Based on our results, we believe that the decrease in the relative abundance of anaerobic bacteria and an increase in the relative abundance of facultative anaerobic bacteria maybe used as risk indexes for female HCC, but are invalid for male HCC.

### Changes in Gut Microbiota Between *LTsc1KO* and Wide-Type *Tsc1^fl/fl^* Mice With the Development of HCC

**FIGURE 4:**
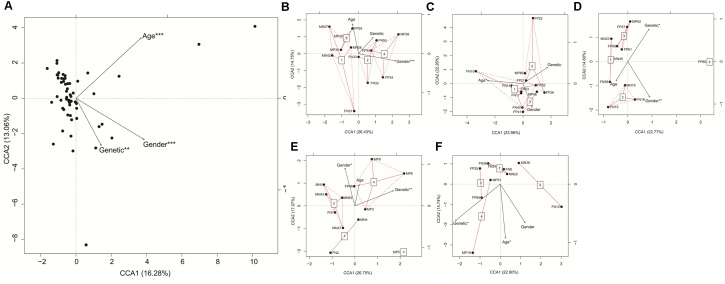
Canonical correspondence analysis (CCA) profiles showed influences of age, gender, and genetic differences on gut microbiota of *LTsc1KO* and wide-type *Tsc1^fl/fl^* mice. *LTsc1KO* mice are a genetic mouse model with liver-specific knockout of the *Tsc1* gene, which causes them to develop spontaneous HCC by 9–10 months of age, and *Tsc1^fl/fl^* mice are their wide-type *Tsc1^fl/fl^* cohorts. The mice were subdivided into five groups according to their age: group 1, age: 68–75 days; group 2, age: 100–133 days; group 3, age: 171–172 days; group 4, age: 185–191 days; and group 5, age: 222–322 days. **(A)** All samples; **(B)** Samples in group 1; **(C)** Samples in group 2; **(D)** Samples in group 3; **(E)** Samples in group 4; **(F)** Samples in group 5. ^∗^*P* < 0.05, ^∗∗^*P* < 0.01, and ^∗∗∗^*P* < 0.001.

CCA based on the detected 498 genera showed that age, sex, and genetic (*LTsc1KO* or wide-type *Tsc1^fl/fl^*) differences significantly influenced the gut microbiota of the mice (**Figure 4A**). In the first two groups (groups 1 and 4), genetic differences did not significantly influence the gut microbiota of the mice. However, in the last three groups, genetic differences significantly influenced the gut microbiota (**Figures 4B–F**).

From the detected 498 genera, 75 genera dominated the fecal microbial communities (their relative abundances were more than 0.1% in at least one sample, Supplementary Figure S3). Together, these genera account for 99.63 ± 0.26% (mean ± standard deviation) of the relative abundance. Genera with relative abundances less than 0.1% were ignored in the further analysis as their abundances would be greatly influenced randomly by sampling error (Ni et al., 2017).

**FIGURE 5:**
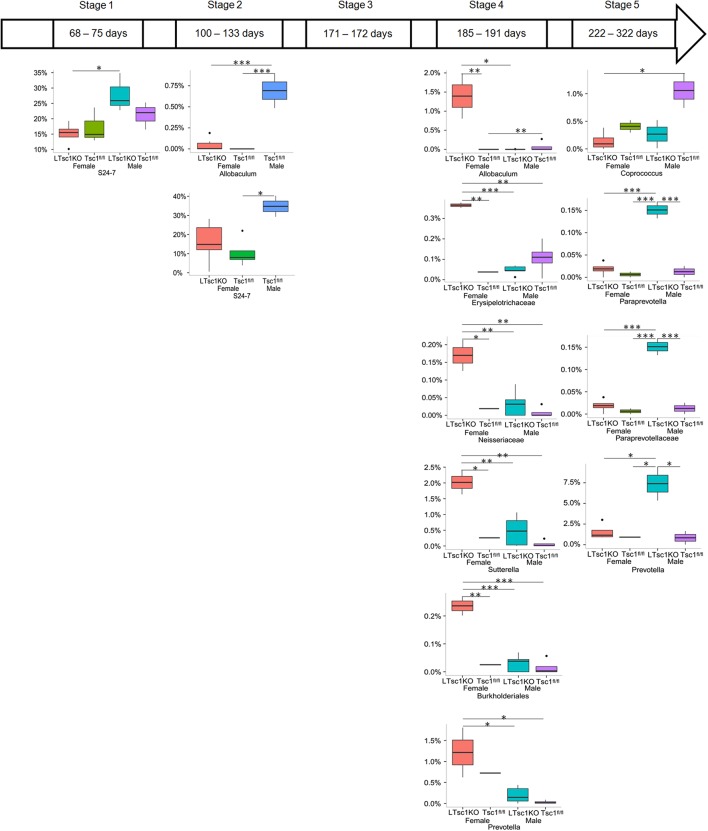
Changes in gut microbiota from *LTsc1KO* and wide-type *Tsc1^fl/fl^* mice at different ages. *LTsc1KO* mice are a genetic mouse model with liver-specific knockout of the *Tsc1* gene, which causes them to develop spontaneous HCC by 9–10 months of age, and *Tsc1^fl/fl^* mice are their wide-type *Tsc1^fl/fl^* cohorts.

At the genus level, the relative abundance of a genus of gram-negative bacterium of the S24-7 family showed significant differences between male and female *LTsc1KO* mice in group 1. In group 2, the relative abundance of gram-positive bacteria belonging to genus *Allobaculum* in male *Tsc1^fl/fl^* mice was significantly higher than that in other cohorts. A genus belonging to the S24-7 family also showed significant difference between male and female *LTsc1KO* mice in group 2. However, in groups 1 and 2, differences in the gut microbiota between *LTsc1KO* and wide-type *Tsc1^fl/fl^* mice were mostly caused by gender differences (**Figure 5**). In group 3, no significant difference for any of the genera was detected between the *LTsc1KO* and wide-type *Tsc1^fl/fl^* mice or between genders. After postnatal 185 days, differences in the gut microbiota between the *LTsc1KO* and wide-type *Tsc1^fl/fl^* mice were obvious. Moreover, the changing patterns were different for the female and male mice (**Figure 5**). For all the genera that showed significant differences in their relative abundance, those from female *LTsc1KO* mice were significant higher than those from others in group 4. However, no other difference was evident, with the exception of gram-positive *Allobaculum* that showed significantly lower abundance in female *Tsc1^fl/fl^* mice than in male mice. The genera showing increased abundances in gut microbiota from female *LTsc1KO* mice in group 4 did not maintain these increased abundances in group 5. The relative abundance of three genera from the Paraprevotellaceae family of gram-negative bacteria was significantly higher in the gut microbiota from male *LTsc1KO* mice than in the other mice (**Figure 5**).

## Discussion

From the perspective of both taxonomic and functional composition, gut microbiota might be linked to and contribute to many complex diseases, including HCC. However, despite considerable progress, most studies have been focused on comparing differences in gut microbiota between patients with HCC and healthy controls. Although differences in phylogenetic and functional compositions have been found, comparison of the differences between patients with HCC and healthy controls cannot determine whether the differences are an inducing factor or a consequence of HCC. Previously, Xie et al. (2016) reported that the composition of the gut microbiota changes significantly in mice responding to STZ-HFD, which is highly relevant to human liver disease. However, the authors did not exclude the continuing influence of STZ-HFD on gut microbiota, given that diet is a major factor affecting the gut microbiota (Faith et al., 2011). Using the *LTsc1KO* model, we were able to successfully exclude the continuing influence of diet on the gut microbiota while describing changes in the gut microbiota during the progression of HCC. Using the *LTsc1KO* model, we found no difference in gut microbiota between *LTsc1KO* and *Tsc1^fl/fl^* mice at postnatal 6 months. However, after postnatal 6 months, differences in gut microbiota between *LTsc1KO* and *Tsc1^fl/fl^* mice emerged before the physiological and biochemical characteristics of the hosts. In addition, female *LTsc1KO* mice exhibited gut microbial disorder earlier than male *LTsc1KO* mice (**Figure 5**). The relatively few genera showing significant differences between *LTsc1KO* and *Tsc1^fl/fl^* mice in our study also implied that diet probably caused false positive differences between STZ-HFD and control mice in previous studies.

Epidemiological studies have provided compelling evidence for the role of sex in liver cancer etiology and survival (Hartwell et al., 2014; Xie et al., 2017). The incidence of newly diagnosed hepatic cancer is 28,410 cases for males and 10,820 cases for females, a 2.6:1 ratio, according to a United States 2016 cancer statistics report (Siegel et al., 2016). The number of deaths from hepatic cancer in 2016 was 18,280 for males and 8,890 for females, a 2.1:1 ratio (Siegel et al., 2016). HCC accounts for 90% of primary liver cancer (Yeh et al., 2013). Xie et al. (2017) reported that sex-dependent effects of gut microbiota regulate hepatic carcinogenic outcomes in a STZ-HFD-induced nonalcoholic steatohepatitis-HCC murine model. Although Menon et al. (2012) detected similar morbidity in both male and female cohorts, in our study, we found that their gut microbiota showed different changing patterns during the development of HCC (**Figure 5**). The female *LTsc1KO* mice exhibited gut microbial disorder earlier than the male *LTsc1KO* mice did. Most of the changes in the gut bacteria were different between female and male *LTsc1KO* mice (**Figure 5**). Moreover, our results also implied that gut microbiota of female mice were more sensitive to liver injury than those of male mice. Even though dysbiosis is considered to promote liver injury and HCC, our results imply that the changes in the gut microbiota probably represented reverse feedback regulation of liver injury and actually prevented the liver injury. This speculation should be verified in future studies.

Previously, *Allobaculum* has been found to be dominant in the intestines of hamsters fed an AIN-93 M diet, and its abundance increased upon grain sorghum lipid extract supplementation, and this abundance correlated with cholesterol metabolic improvement (Miriam and Buenviaje, 1989). The blood cholesterol-lowering effect of alginate has also been reported, which might be associated with its fermentation properties (Kuda et al., 1997). However, the reason for the increase in the relative abundance of *Allobaculum* species in the *LTsc1KO* mice in group 4 is unclear. Except *Allobaculum* (a gram-positive bacterial genus; An et al., 2013), all the other genera showing significant changes contained gram-negative bacteria, and several pathogenic or disease-related bacteria have been previously reported to belong to these genera (van Ulsen and Tommassen, 2006; Sakon et al., 2008). For instance, Erysipelotrichaceae species have shown changes in their abundance in patients with inflammatory diseases (Kaakoush, 2015). In our study as well, the relative abundance of Erysipelotrichaceae species was found to be significantly increased in the gut microbiota of female *LTsc1KO* mice in group 4. An increase in the abundance of *Sutterella* species has also observed in the feces of children with autism spectrum disorder (Wang et al., 2013). Burkholderiales bacteria are especially dangerous for intensive care unit patients and patients with chronic lung diseases (Voronina et al., 2015). Therefore, the increases in the abundance of *Allobaculum*, Erysipelotrichaceae, Neisseriaceae, *Sutterella*, Burkholderiales, and *Prevotella* species might have potential for application as indicators of risk of female HCC. Although Paraprevotellaceae species have been commonly correlated with diet (Fernandes et al., 2014; Li et al., 2015), they have also been reported to be correlated with diseases, especially those caused by *Prevotella* species (Gharbia et al., 1994; Brook et al., 1995). Therefore, an increased abundance of Parapervotella, Paraprevotellaceae, and *Prevotella* species can be applied as indicators of risk of male HCC.

An increase in the relative abundance of gram-negative bacteria in the gut microbiota could increase the concentrations of endotoxins in the plasma and the liver (Xie et al., 2016). Several lines of evidence indicate that LPS accumulation contributes to the pathogenesis of HCC by eliciting proinflammatory responses in the liver (Yu et al., 2010). However, our results showed no significant difference was detected between the relative abundance of gram-negative or gram-positive bacteria from the *LTsc1KO* and *Tsc1^fl/fl^* mice in any of the groups (Supplementary Figures S2D,E). For LPS metabolism, no significant difference was detected in the relative abundance of the genes that participate in the KEGG pathways of LPS biosynthesis, LPS transport system, or LPS export system between the *LTsc1KO* and *Tsc1^fl/fl^* mice in any of the groups (Supplementary Figures S2F–H). These results imply that STZ-HFD might be the cause of the relative abundance of gram-negative bacteria and endotoxins, as HFD could significantly increase the relative abundance of gram-negative bacteria. This shows that the *LTsc1KO* model is an ideal model for studying the relationship between gut microbiota and the development of HCC, which excludes the influence of diet and other inducers.

The gut microbiota is influenced by diverse endogenous and exogenous factors, such as host development stage, diet components, health condition and many as-yet uncharacterized factors (Hooper and Gordon, 2001; Muegge et al., 2011; Ni et al., 2014; Yan et al., 2016; Li et al., 2017). This leads to obvious differences in the microbiota between host individuals or between different habitats (Costello et al., 2009), which in turn increases the difficulty of detecting significant differences. Therefore, many reports broaden the level of significant difference from 0.05 to 0.10 when detecting whether a specific factor significantly influences the microbiota (Ross et al., 2004; Xu et al., 2014; Zijlmans et al., 2015). In addition, although the *LTsc1KO* model is an ideal model for studying the relationship between gut microbiota and the development of HCC, our study had limitations of sample quantities and some liver biochemical and histological characteristics of the mice with different ages were deficient; moreover, only one sample or sometimes no samples were collected in some sample groups. These might be the reasons why the genera showing increased abundance in the gut microbiota from female *LTsc1KO* mice in group 4 did not maintain the increased abundance in group 5. Therefore, the causality between the changes in gut microbial composition and the development of HCC needs to be further verifies using germ-free mice. Moreover, the internal mechanisms causing changes in the gut microbiota and the effect of these changes on the development HCC should be study in more detail in the future.

Nevertheless, we detected different changing patterns between female and male *LTsc1KO* mice of different ages in our study with the development of HCC. Our results also suggest that the decrease in the relative abundance of anaerobic bacteria and the increase in the relative abundance of facultative anaerobic bacteria have potential for use as risk indexes of female HCC. The increased abundances of *Allobaculum*, Erysipelotrichaceae, Neisseriaceae, *Sutterella*, Burkholderiales, and *Prevotella* species might also be applied as risk indicators of female HCC, and for male HCC, increased abundances of Paraprevotella, Paraprevotellaceae, and *Prevotella* species might be potential risk indicators.

## Author Contributions

RH, JN, and YiG designed the experiments. RH, TL, XB, YL, and YaG performed the experiments. RH, JN, and PZ analyzed the data. JN and YiG wrote the paper. All authors reviewed and edited the manuscript.

## Conflict of Interest Statement

The authors declare that the research was conducted in the absence of any commercial or financial relationships that could be construed as a potential conflict of interest. The reviewer AA and handling Editor declared their shared affiliation.
