# Supplementary material for: Different Sex-Based Responses of Gut Microbiota During the Development of Hepatocellular Carcinoma in Liver-Specific Tsc1-Knockout Mice
Source: Front Microbiol. 2018 May 16;9:1008. doi: 10.3389/fmicb.2018.01008 (PMC5964185; doi:10.3389/fmicb.2018.01008)

Supplementary Materials

Different Sex-based Responses of Gut Microbiota during the Development of Hepatocellular Carcinoma in Liver-specific *Tsc1*-Knockout Mice

Rong Huang[1,2#], Ting Li[1,2#], Jiajia Ni[1,2#], Xiaochun Bai[3], Yi Gao[1,2*], Yang Li[1,2], Peng Zhang[1,2], Yan Gong[3]

[1]Department of Hepatobiliary Surgery II, Guangdong Provincial Research Center of Artificial Organ and Tissue Engineering, Zhujiang Hospital of Southern Medical University, Guanzhou, 510280, China
[2]State Key Laboratory of Organ Failure Research, Southern Medical University, Guangzhou, 510515, China
[3]Department of Cell Biology, School of Basic Medical Science, Southern Medical University, Guangzhou, 510515, China

[#]These authors contributed equally to this work.

*Correspondence
Yi Gao
gaoyi6146@163.com

Table S1 Sample information.

SampleID	Stage	Age (days)	SequenceFileName	Gender	GeneticTreatment	BirthDate	SamplingData
LTsc1KO-F-2-1	2	114	FN10	Female (F)	LTsc1KO	Sep.6	Dec.29
LTsc1KO-F-2-2	2	114	FN11	Female (F)	LTsc1KO	Sep.6	Dec.29
LTsc1KO-F-5-3	5	272	FN12	Female (F)	LTsc1KO	Apr.1	Dec.29
LTsc1KO-F-3-1	3	171	FN15	Female (F)	LTsc1KO	Jul.11	Dec.29
LTsc1KO-F-3-2	3	171	FN16	Female (F)	LTsc1KO	Jul.11	Dec.29
LTsc1KO-F-2-3	2	103	FN17	Female (F)	LTsc1KO	Sep.17	Dec.29
LTsc1KO-F-4-1	4	186	FN2	Female (F)	LTsc1KO	Jun.26	Dec.29
LTsc1KO-F-1-1	1	75	FN31	Female (F)	LTsc1KO	Oct.15	Dec.29
LTsc1KO-F-1-2	1	75	FN32	Female (F)	LTsc1KO	Oct.15	Dec.29
LTsc1KO-F-1-3	1	70	FN33	Female (F)	LTsc1KO	Oct.20	Dec.29
LTsc1KO-F-5-1	5	222	FN36	Female (F)	LTsc1KO	May.21	Dec.29
LTsc1KO-F-5-2	5	222	FN37	Female (F)	LTsc1KO	May.21	Dec.29
LTsc1KO-F-2-4	2	103	FN40	Female (F)	LTsc1KO	Sep.17	Dec.29
LTsc1KO-F-2-5	2	133	FN52	Female (F)	LTsc1KO	Aug.18	Dec.29
LTsc1KO-F-2-6	2	133	FN53	Female (F)	LTsc1KO	Aug.18	Dec.29
LTsc1KO-F-1-4	1	68	FN55	Female (F)	LTsc1KO	Oct.22	Dec.29
LTsc1KO-F-3-3	3	171	FN58	Female (F)	LTsc1KO	Jul.11	Dec.29
LTsc1KO-F-4-2	4	186	FN7	Female (F)	LTsc1KO	Jun.26	Dec.29
LTsc1KO-F-5-4	5	272	FN8	Female (F)	LTsc1KO	Apr.1	Dec.29
$Tsc1^{fl/fl}$-F-2-1	2	114	FP21	Female (F)	$Tsc1^{fl/fl}$	Sep.6	Dec.29
$Tsc1^{fl/fl}$-F-2-2	2	114	FP22	Female (F)	$Tsc1^{fl/fl}$	Sep.6	Dec.29
$Tsc1^{fl/fl}$-F-5-1	5	222	FP25	Female (F)	$Tsc1^{fl/fl}$	May.21	Dec.29
$Tsc1^{fl/fl}$-F-1-1	1	75	FP34	Female (F)	$Tsc1^{fl/fl}$	Oct.15	Dec.29
$Tsc1^{fl/fl}$-F-1-2	1	70	FP35	Female (F)	$Tsc1^{fl/fl}$	Oct.20	Dec.29
$Tsc1^{fl/fl}$-F-2-3	2	114	FP39	Female (F)	$Tsc1^{fl/fl}$	Sep.6	Dec.29
$Tsc1^{fl/fl}$-F-2-4	2	100	FP41	Female (F)	$Tsc1^{fl/fl}$	Sep.20	Dec.29
$Tsc1^{fl/fl}$-F-4-1	4	186	FP46	Female (F)	$Tsc1^{fl/fl}$	Jun.26	Dec.29
$Tsc1^{fl/fl}$-F-1-3	1	68	FP54	Female (F)	$Tsc1^{fl/fl}$	Oct.22	Dec.29
$Tsc1^{fl/fl}$-F-3-1	3	172	FP57	Female (F)	$Tsc1^{fl/fl}$	Jul.10	Dec.29
$Tsc1^{fl/fl}$-F-3-2	3	171	FP59	Female (F)	$Tsc1^{fl/fl}$	Jul.11	Dec.29
$Tsc1^{fl/fl}$-F-3-3	3	171	FP61	Female (F)	$Tsc1^{fl/fl}$	Jul.11	Dec.29
$Tsc1^{fl/fl}$-F-5-2	5	322	FP64	Female (F)	$Tsc1^{fl/fl}$	Feb.11	Dec.29
$Tsc1^{fl/fl}$-F-3-4	3	171	FP65	Female (F)	$Tsc1^{fl/fl}$	Jul.11	Dec.29
LTsc1KO-M-4-1	4	185	MN1	Male (M)	LTsc1KO	Jun.27	Dec.29
LTsc1KO-M-3-1	3	172	MN18	Male (M)	LTsc1KO	Jul.10	Dec.29
LTsc1KO-M-5-1	5	222	MN20	Male (M)	LTsc1KO	May.21	Dec.29
LTsc1KO-M-3-2	3	171	MN23	Male (M)	LTsc1KO	Jul.11	Dec.29
LTsc1KO-M-1-1	1	75	MN26	Male (M)	LTsc1KO	Oct.15	Dec.29
LTsc1KO-M-1-2	1	70	MN27	Male (M)	LTsc1KO	Oct.20	Dec.29
LTsc1KO-M-5-2	5	315	MN38	Male (M)	LTsc1KO	Feb.18	Dec.29
LTsc1KO-M-4-2	4	185	MN4	Male (M)	LTsc1KO	Jun.27	Dec.29
LTsc1KO-M-1-3	1	70	MN42	Male (M)	LTsc1KO	Oct.20	Dec.29
LTsc1KO-M-4-3	4	185	MN43	Male (M)	LTsc1KO	Jun.27	Dec.29
LTsc1KO-M-3-3	3	172	MN45	Male (M)	LTsc1KO	Jul.10	Dec.29
LTsc1KO-M-4-4	4	190	MN47	Male (M)	LTsc1KO	Jun.22	Dec.29

LTsc1KO-M-4-5	4	185	MN63	Male (M)	LTsc1KO	Jun.27	Dec.29
$Tsc1^{fl/fl}$-M-5-1	5	322	MP19	Male (M)	$Tsc1^{fl/fl}$	Feb.11	Dec.29
$Tsc1^{fl/fl}$-M-1-1	1	70	MP28	Male (M)	$Tsc1^{fl/fl}$	Oct.20	Dec.29
$Tsc1^{fl/fl}$-M-1-2	1	70	MP29	Male (M)	$Tsc1^{fl/fl}$	Oct.20	Dec.29
$Tsc1^{fl/fl}$-M-4-1	4	191	MP3	Male (M)	$Tsc1^{fl/fl}$	Jun.21	Dec.29
$Tsc1^{fl/fl}$-M-1-3	1	70	MP30	Male (M)	$Tsc1^{fl/fl}$	Oct.20	Dec.29
$Tsc1^{fl/fl}$-M-2-1	2	133	MP48	Male (M)	$Tsc1^{fl/fl}$	Aug.18	Dec.29
$Tsc1^{fl/fl}$-M-4-2	4	185	MP5	Male (M)	$Tsc1^{fl/fl}$	Jun.27	Dec.29
$Tsc1^{fl/fl}$-M-5-2	5	315	MP51	Male (M)	$Tsc1^{fl/fl}$	Feb.18	Dec.29
$Tsc1^{fl/fl}$-M-4-3	4	185	MP6	Male (M)	$Tsc1^{fl/fl}$	Jun.27	Dec.29
$Tsc1^{fl/fl}$-M-3-1	3	171	MP62	Male (M)	$Tsc1^{fl/fl}$	Jul.11	Dec.29
$Tsc1^{fl/fl}$-M-2-2	2	105	MP66	Male (M)	$Tsc1^{fl/fl}$	Sep.15	Dec.29
$Tsc1^{fl/fl}$-M-4-4	4	190	MP9	Male (M)	$Tsc1^{fl/fl}$	Jun.22	Dec.29

Figure S1 Alpha-diversity of gut microbiota from *LTsc1KO* and wide-type $Tsc1^{fl/fl}$ mice with different days of age. *LTsc1KO* mice are a genetic mouse model with liver-specific knockout of the *Tsc1* gene, which causes them to develop spontaneous hepatocellular carcinoma (HCC) by 9–10 months of age, and the $Tsc1^{fl/fl}$ mice are their wide-type $Tsc1^{fl/fl}$ cohorts. The mice were subdivided into 5 groups according to their age: group 1, age: 68–75 days; group 2, age: 100–133 days; group 3, age: 171–172 days; group 4, age: 185–191days; and group 5, age: 222–322 days. FN, female *LTsc1KO* mice; MN, male *LTsc1KO* mice; FP, female $Tsc1^{fl/fl}$ mice; and MP, male $Tsc1^{fl/fl}$ mice.

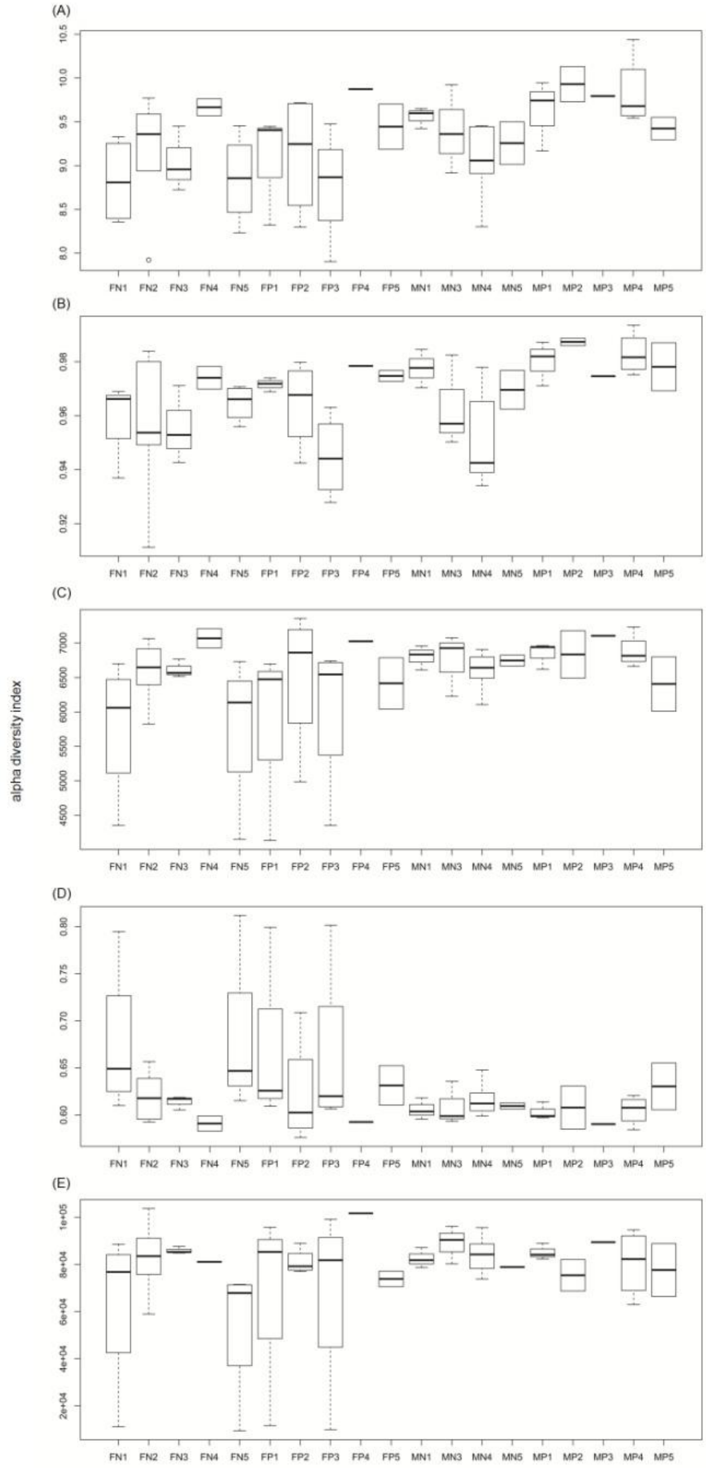

Figure S2. Relative abundance of aerobic bacteria (A), anaerobic bacteria (B), facultative anaerobic bacteria (C), gram-negative bacteria (D), gram-positive bacteria (E), the genes that participate in the KEGG pathway of lipopolysaccharide (LPS) biosynthesis (F), genes that participate in the KEGG pathway of LPS transport system (G), and genes that participate in the KEGG pathway of LPS export system (H) in fecal microbiota from *LTsc1KO* and *Tsc1*$^{fl/fl}$ mice. *LTsc1KO* mice are a genetic mouse model with liver-specific knockout of the *Tsc1* gene, which causes them to develop spontaneous hepatocellular carcinoma (HCC) by 9–10 months of age, and the *Tsc1*$^{fl/fl}$ mice are their wide-type *Tsc1*$^{fl/fl}$ cohorts. The mice were subdivided into 5 groups according to their age: group 1, age: 68–75 days; group 2, age: 100–133 days; group 3, age: 171–172 days; group 4, age: 185–191days; and group 5, age: 222–322 days.

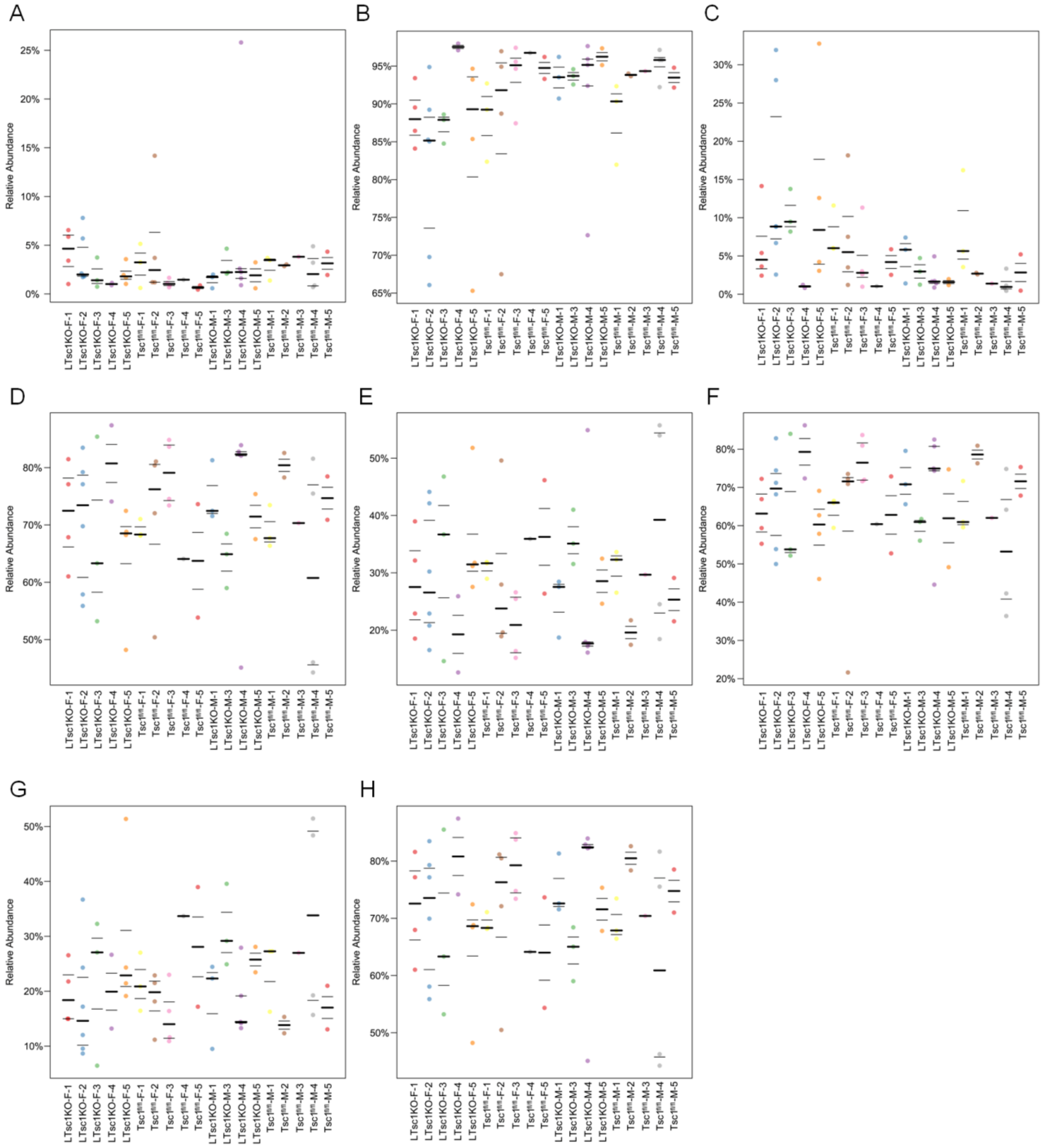

Figure S3. Heatmap of gut microbiota from *LTsc1KO* and wide-type *Tsc1$^{fl/fl}$* mice with different days of age. *LTsc1KO* mice are a genetic mouse model with liver-specific knockout of the *Tsc1* gene, which causes them to develop spontaneous hepatocellular carcinoma (HCC) by 9–10 months of age, and the *Tsc1$^{fl/fl}$* mice are their wide-type *Tsc1$^{fl/fl}$* cohorts. The mice were subdivided into 5 groups according to their age: group 1, age: 68–75 days; group 2, age: 100–133 days; group 3, age: 171–172 days; group 4, age: 185–191days; and group 5, age: 222–322 days.

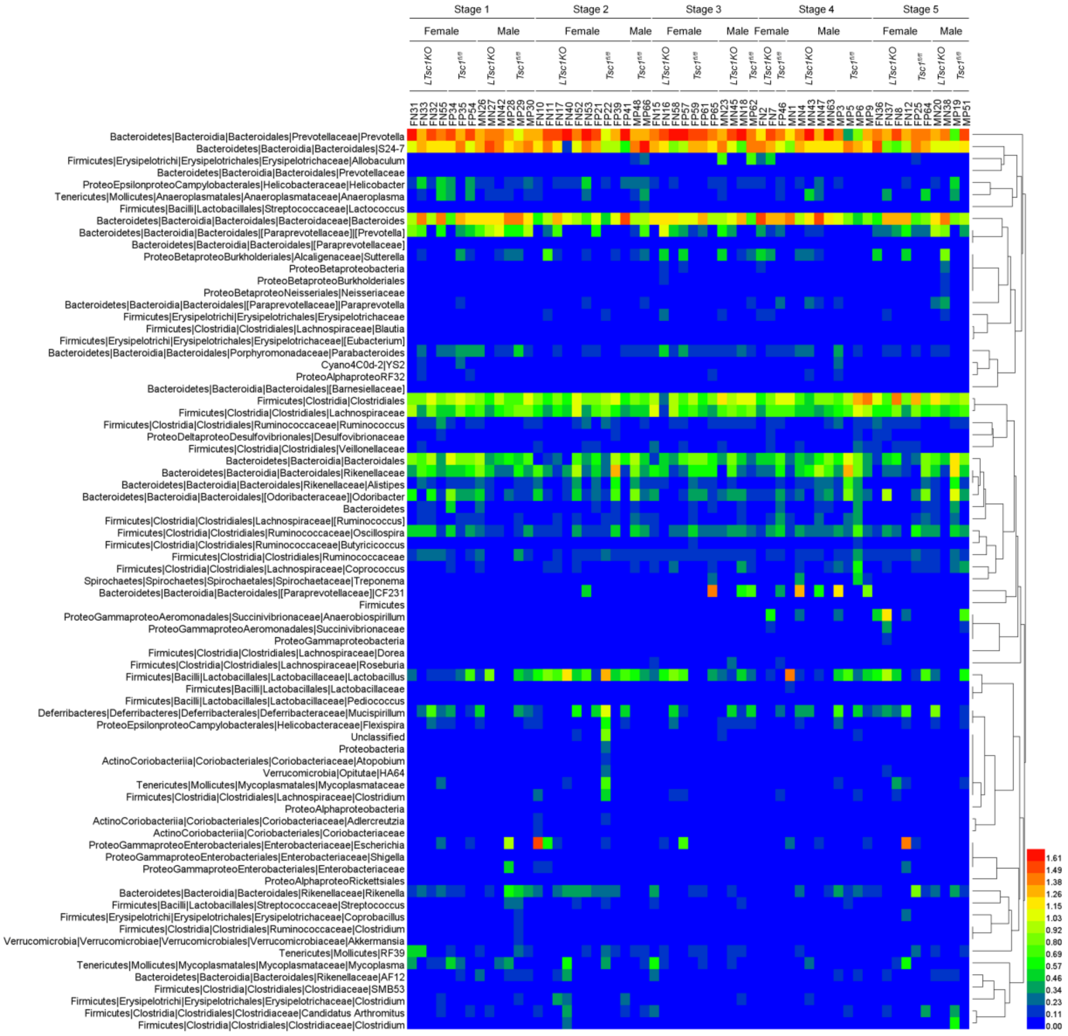

Supplement: Supplementary file 1 [file Presentation_1.PDF]
